# Ventriculo-atrial defect after bioprosthetic aortic valve replacement

**DOI:** 10.1186/s13019-014-0137-1

**Published:** 2014-10-02

**Authors:** Jayant S Jainandunsing, Remco Bergman, Jacob Wilkens, Angela Wang, Guido Michielon, Ehsan Natour

**Affiliations:** Department of Anesthesia and Pain Medicine, University of Groningen, University Medical Center Groningen, Hanzeplein 1, Groningen, 9700 RB The Netherlands; Department of Cardiothoracic Surgery, University of Groningen, University Medical Center Groningen, Hanzeplein 1, Groningen, 9700 RB The Netherlands; Department of Anesthesia Critical Care and Pain Medicine, Beth Israel Deaconess Medical Center, Harvard Medical School, 330 Brookline Avenue, Boston, 02215 Massachussetts USA; Department Cardiothoracic Surgery, University Medical Center Groningen, Hanzeplein 1, Groningen, 9700 RB The Netherlands

**Keywords:** Ventriculo-atrial defect, Echocardiography, Gerbode defect, Aortic valve

## Abstract

We present a case of a 71-year-old Caucasian male with a ventriculo-atrial defect due to infective endocarditis, originating from his aortic root near a bioprosthetic aortic valve, implanted 4 years earlier. Ventriculo-atrial defects are rare and can occur after endocarditis with abscess formation, usually in native tissue. We report a ventriculo-atrial defect due to a paravalvular aortic prosthetic defect, secondary to inflammation, a novel third type of a Gerbode defect. Case presentation, clinical decision making and surgical approach are discusses in this report.

**Electronic supplementary material:**

The online version of this article (doi:10.1186/s13019-014-0137-1) contains supplementary material, which is available to authorized users.

## Background

Ventriculo-atrial defects are rare defects, first described in 1958 by Gerbode [1]. These defects until now were subdivided into two types Type-1 Gerbode defect is an acquired defect through the ventriculo-atrial membranous septum, resulting in a direct left ventricle to right atrium shunt. Type-2 Gerbode defect is an indirect congenital defect, there are two defects present, a ventricle septum defect and a defect in the tricuspid septal leaflet, thus creating an indirect left to right shunt. In this paper we present a case of a third type Gerbode defect, a peri-prosthetic defect with a left-to right shunt due to prosthesis dehiscence, creating a direct shunt from the left ventricle and aortic annulus into the right atrium. Acquired ventriculo-atrial defects can occur due to infectious endocarditis, ischemic heart disease or following tricuspid or mitral valve surgery [2]-[4]. On echocardiographic examination a systolic high-pressure jet can usually be detected, caused by the defect. This jet is sometimes misinterpreted as severe asymmetrical tricuspid regurgitation, wrongly suggesting increased pulmonary pressure [5],[6]. In this report we present a successful surgical treatment of an acquired ventriculo-atrial defect, tricuspid insufficiency, anterior mitral leaflet and bioprosthetic aortic valve endocarditis.

## Case presentation

A 71-year-old Caucasian male, with a history of aortic valve replacement in 2010 (Carpentier Edwards Perimount Magna Ease® stented bioprothesis, Ø 27 mm), was one year later referred to our academic center, with symptoms of fever (38.5°C) and malaise after two weeks of unsuccessful treatment (Amoxicillin) for suspected urinary-tract infection.

**Figure 1:**
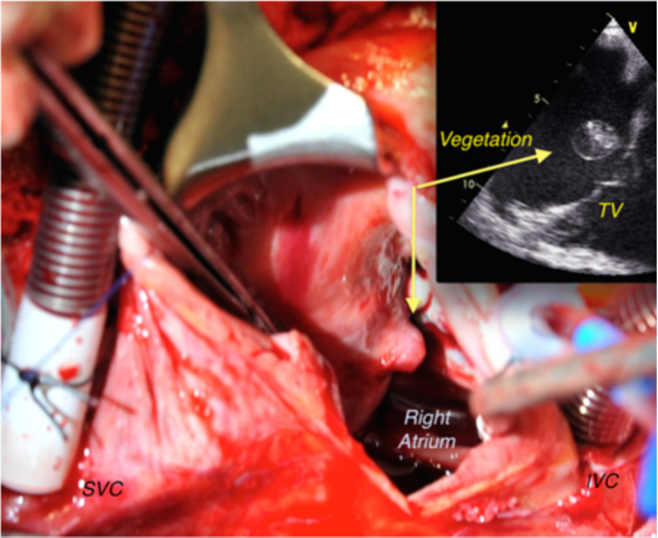
**Vegetation can be seen at the tricuspid annulus near the septal leaflet.** Echocardigraphy suggests tricuspid leaflet involvement, however the intra-operative image shows the vegetation on the atrial septal wall.

**Figure 2:**
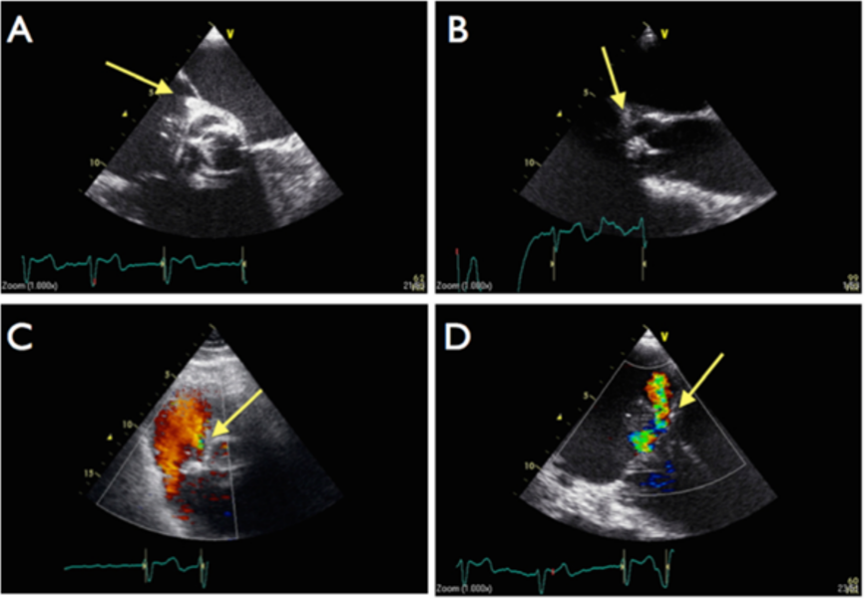
**Transesophageal echocardiograpy views.** Panel A: Mid-esophageal RV inflow/outflow view shows a paravalvular abcess near the atrial septum and involvment of the tricuspid valve insertion (arrow). Panel **B**: Mide-Esophageal long axis view showing vegetation on the aortic valve prosthesis. Panel **C**: zoomed in on the atrial septum, showing the Gerbode like defect with turblent flow (arrow). Panel **D**: severe tricuspid insufficiency (arrow).

A trans-thoracic echocardiographic examination was performed it revealed mild tricuspid regurgitation and an mobile echo density with clear delineated borders, adjacent to the tricuspid valve, raising the suspicion for infective endocarditis. Nine days after admission patient suddenly developed a 3rd degree atrioventricular block and signs of forward cardiac failure. Transesophageal echocardiography (TEE) revealed a mobile structure near the septal leaflet of the tricuspid valve, grade III Tricuspid Regurgitation (TR) and a suspected abscess of the aortic annulus at the level of the Non Coronary Cusp (NCC) (Figures 1 and 2).

The need for external pacing with increasing systemic malperfusion (oliguria), combined with a positive response to antibiotics, signified by dropping infectious parameters led to the decision for surgical treatment at this stage.

### Surgical procedure

**Figure 3:**
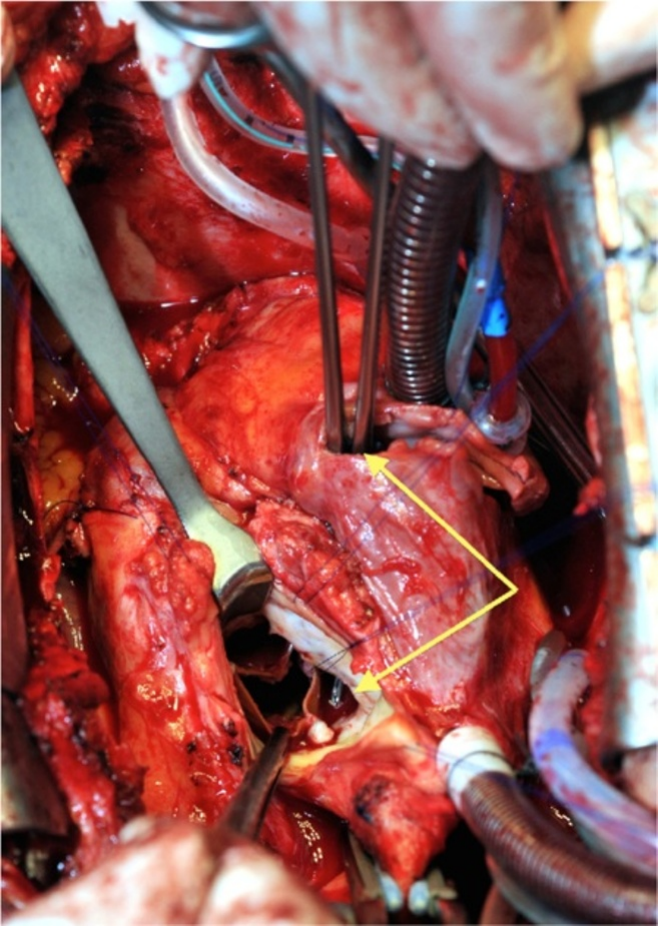
**Intraoperative image showing a clamp going into the right atrium (arrows) and exiting in the left ventricle (the defect runs from subvalvular to epi-annular).**

After induction of anesthesia, sternotomy and careful dissection of the severe adhesions it was noted that the aortic ascending aortic arch was dilated (Ø 5 cm). After initiation of cardio pulmonary bypass the aorta was clamped and combined antegrade & retrograde cold blood cardioplegia was given. After aortotomy the previously implanted aortic valve was noted to have vegetations most prominently on the non-coronary cusp. A paravalvular abscess cavity was found, which extended subvalvular into the left ventricle and continued to the right atrium, Gerbode-like defect (Figure 3). After atriotomy inspection of the Tricuspid valve revealed vegetations of the septal leaflet and a severe annular dilation (>40 mm) causing severe Tricuspid regurgitation. After explantation of the aortic prosthetic valve, vegetations were seen on the Anterior Mitral Leaflet (AML). A partial resection of the base of the AML was also performed. Reconstruction of the aorto-mitral continuity was done with a pericardial patch using single stitch technique.

**Figure 4:**
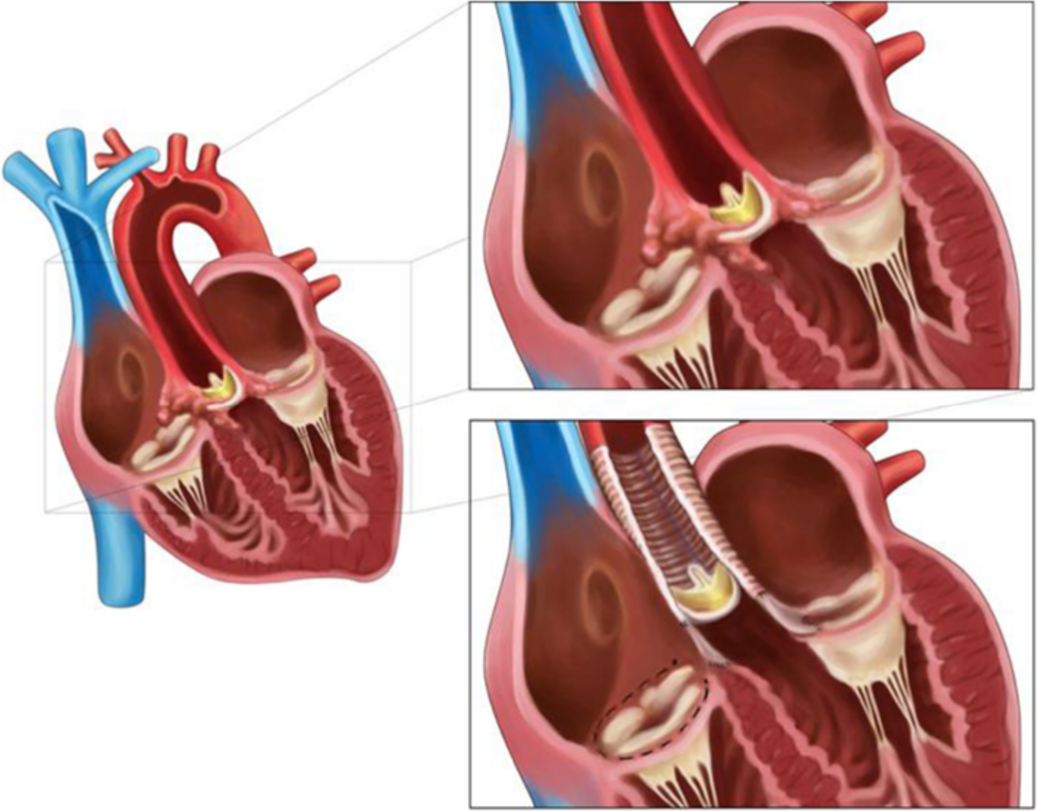
**Schematic images of the situation before and after surgery, top right, shows the Gerbode-like defect and initial prosthetic valve.** Bottom right shows the situation after surgery with the Vascutek prosthesis, new biological valve, reconstruction of the Aorto-Mitral continuity and the DeVega annuloplasty.

The aortic root and dilated ascending aorta were reconstructed with a Vascutec® prosthesis (Ø 26 mm Vascutec Vascular prosthesis, Glasgow, United Kingdom). The bioprosthetic aortic valve was replaced by a new stentless aortic bioprosthesis. Tricuspid annuloplasty was performed using the DeVega technique. Finally the Gerbode defect was closed with felt pledges and sutures (Figure 4).

TEE examination showed adequate pump function at the end of surgery. It also showed normal function of the mitral and aortic valves and only grade I Tricuspid regurgitation. After achieving adequate hemostasis, closure was performed in the standard fashion and the patient was transported to the ICU in a hemodynamically stable condition. Recovery was uneventful, patient was discharged in a good clinical condition.

## Conclusions

Our patient had a ventriculo-atrial defect due to infective endocarditis, valve cultures were positive for streptococcus agalactiae, originating from the aortic root adjacent to his bioprosthetic aortic valve, implanted 1 year earlier.

The diagnosis of infective endocarditis is usually made according to the Duke criteria [7]. Based on a combination of minor and major criteria to confirm endocarditis, as a possible diagnosis or reject it. In our case positive blood cultures, fever and predisposition (artificial valve), together with findings on TEE, confirmed the diagnosis. Infective endocarditis is a serious illness with mortality rates ranging from 9.6-26% [8]-[10].

Mainstay of treatment for infective endocarditis is antibiotic treatment, patient was treated with penicillin. Surgical treatment is reserved for treatment of complications due to degenerative disease process or resistant micro-organisms [11]. The indications for surgery can be divided into heart failure, uncontrolled infection and prevention of embolism [11]. This case represents a severe endocarditis; not only did our patient have heart failure (as evidenced by the oliguria and need for pacing) in addition there was uncontrolled infection (paravalvular abscess) as well as fistula formation leading to hemodynamic compromise. Atrioventricular conduction runs through fibers in the atrioventricular septum our patient developed a 3rd degree AV block indicating inter-atrial septum conductive pathway involvement. Cause of which was likely the formation of the paravalvular abscess.

During the surgical procedure the extent of the damage was greater than initially anticipated with involvement of not only the aortic, but also the tricuspid and mitral valve. The Gerbode defect was not seen initially on echocardiography due to the existence of severe tricuspid regurgitation hiding the jet from the left ventricle entering the right atrium.

**Figure 5:**
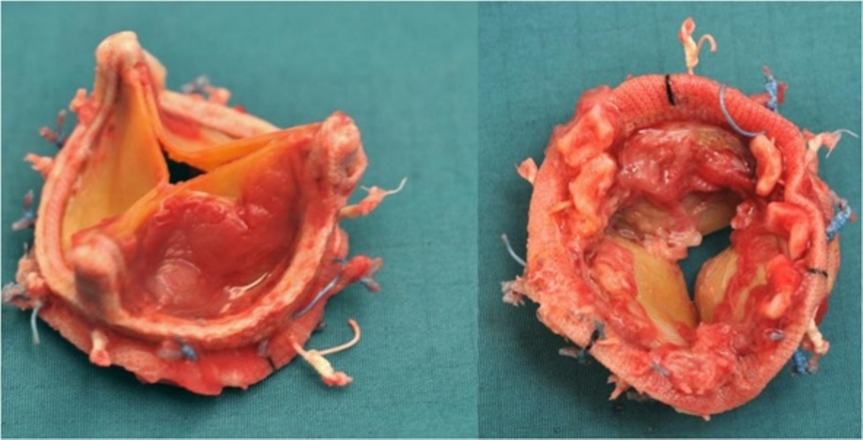
**Aortic valve prosthesis after explantation with severe vegetations.**

Decision to proceed to surgery was based on the suspicion of infective endocarditis with clinical deterioration despite antibiotic treatment. Combination of forward cardiac failure together with arrhythmia ultimately dictated surgical management. In retrospect the peculiar object seen in the right atrium (Figure 1) and vegetations on the bioprosthesis (Figure 5) could have been seen as evidence of septal involvement.

Ventriculo-atrial defects are complex entities, which require a thorough understanding of pathophysiology. Echocardiographic images should be looked at with great care since inter-septal jets can be mistaken for valve insufficiencies, and the true extent of valvular involvement can be underestimated. Patients with endocarditis are continuously at risk for severe complications, heart failure, sepsis and abscess formation. Rapid decision-making should involve a multi-disciplinary approach. These complex cases need to be addressed with utmost care in experienced clinical centers.

## Consent

Written informed consent was obtained from the patient for publication of this case report and any accompanying images. A copy of the written consent is available for review by the Editor-in-Chief of this journal.

## Authors’ original submitted files for images

Below are the links to the authors’ original submitted files for images. Authors’ original file for figure 1 Authors’ original file for figure 2 Authors’ original file for figure 3 Authors’ original file for figure 4 Authors’ original file for figure 5

## Abbreviations

TEE: Transesophageal echocardiography
TR: Tricuspid regurgitation (TR)
NCC: Non coronary cusp (NCC)
ICU: Intensive care unit
