# Erratum: Ventriculo-atrial defect after bioprosthetic aortic valve replacement

**DOI:** 10.1186/s13019-015-0279-9

**Published:** 2015-05-29

**Authors:** Jayant S. Jainandunsing, Remco Bergman, Jacob Wilkens, Angela Wang, Guido Michielon, Ehsan Natour

**Affiliations:** 1 Department of Anesthesia and Pain Medicine, University of Groningen, University Medical Center, Groningen, The Netherlands; 2 Department of Cardiothoracic Surgery, University of Groningen, University Medical Center Groningen, Hanzeplein 1, 9700 RB Groningen, The Netherlands; 3 Department of Anesthesia Critical Care and Pain Medicine, Beth Israel Deaconess Medical Center, Harvard Medical School, 330 Brookline Avenue, Boston, Massachussetts 02215 USA

After publication of the article [1] it was discovered that this manuscript was mistakenly given a duplicated citation number. The correct citation of 9:199 has now been updated in all versions of this manuscript. We apologise for any inconvenience caused by this error.
