# Supplementary figures and images for: Ventriculo-atrial defect after bioprosthetic aortic valve replacement
Source: J Cardiothorac Surg. 2014 Oct 2;9:137. doi: 10.1186/s13019-014-0137-1 (PMC4205768; doi:10.1186/s13019-014-0137-1)

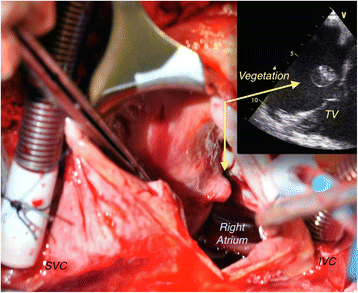

Supplement: Supplementary file 1 — Authors’ original file for figure 1 [file 13019_2014_137_MOESM1_ESM.gif]

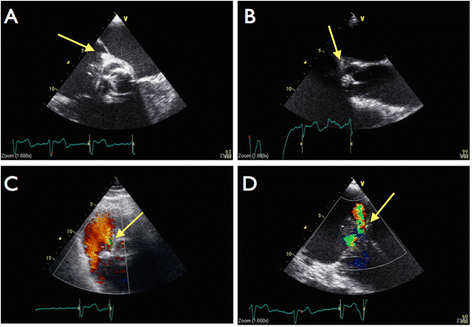

Supplement: Supplementary file 2 — Authors’ original file for figure 2 [file 13019_2014_137_MOESM2_ESM.gif]

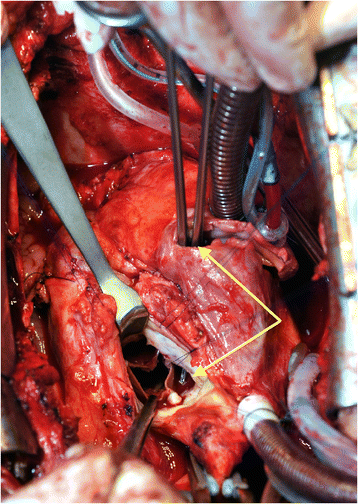

Supplement: Supplementary file 3 — Authors’ original file for figure 3 [file 13019_2014_137_MOESM3_ESM.gif]

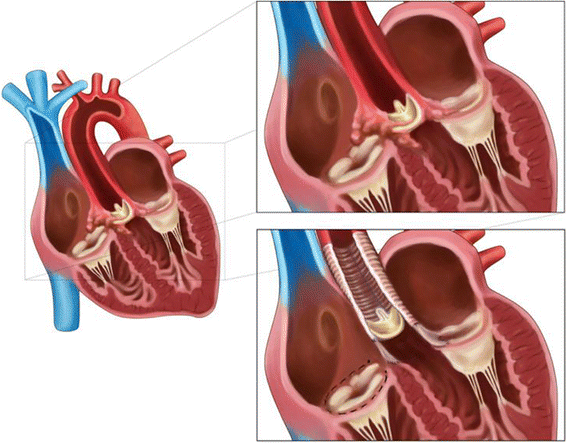

Supplement: Supplementary file 4 — Authors’ original file for figure 4 [file 13019_2014_137_MOESM4_ESM.gif]

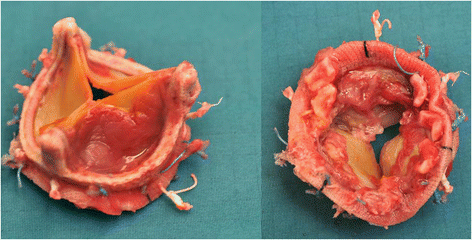

Supplement: Supplementary file 5 — Authors’ original file for figure 5 [file 13019_2014_137_MOESM5_ESM.gif]
